# Malectin Participates in a Backup Glycoprotein Quality Control Pathway in the Mammalian ER

**DOI:** 10.1371/journal.pone.0016304

**Published:** 2011-01-26

**Authors:** Carmela Galli, Riccardo Bernasconi, Tatiana Soldà, Verena Calanca, Maurizio Molinari

**Affiliations:** 1 Institute for Research in Biomedicine, Bellinzona, Switzerland; 2 School of Life Sciences, Ecole Polytechnique Fédérale de Lausanne, Lausanne, Switzerland; Fred Hutchinson Cancer Research Center, United States of America

## Abstract

Malectin is a conserved, endoplasmic reticulum (ER)-resident lectin that recognizes high mannose oligosaccharides displaying terminal glucose residues. Here we show that Malectin is an ER stress-induced protein that selectively associates with glycopolypeptides without affecting their entry and their retention in the Calnexin chaperone system. Analysis of the *obligate* Calnexin client influenza virus hemagglutinin (HA) revealed that Calnexin and Malectin associated with different timing to different HA conformers and that Malectin associated with misfolded HA. Analysis of the *facultative* Calnexin clients NHK and α1-antitrypsin (α1AT) revealed that induction of Malectin expression to simulate conditions of ER stress resulted in persistent association between the ER lectin and the model cargo glycoproteins, interfered with processing of cargo-linked oligosaccharides and reduced cargo secretion. We propose that Malectin intervention is activated upon ER stress to inhibit secretion of defective gene products that might be generated under conditions of aberrant functioning of the ER quality control machinery.

## Introduction

**Figure 1:**
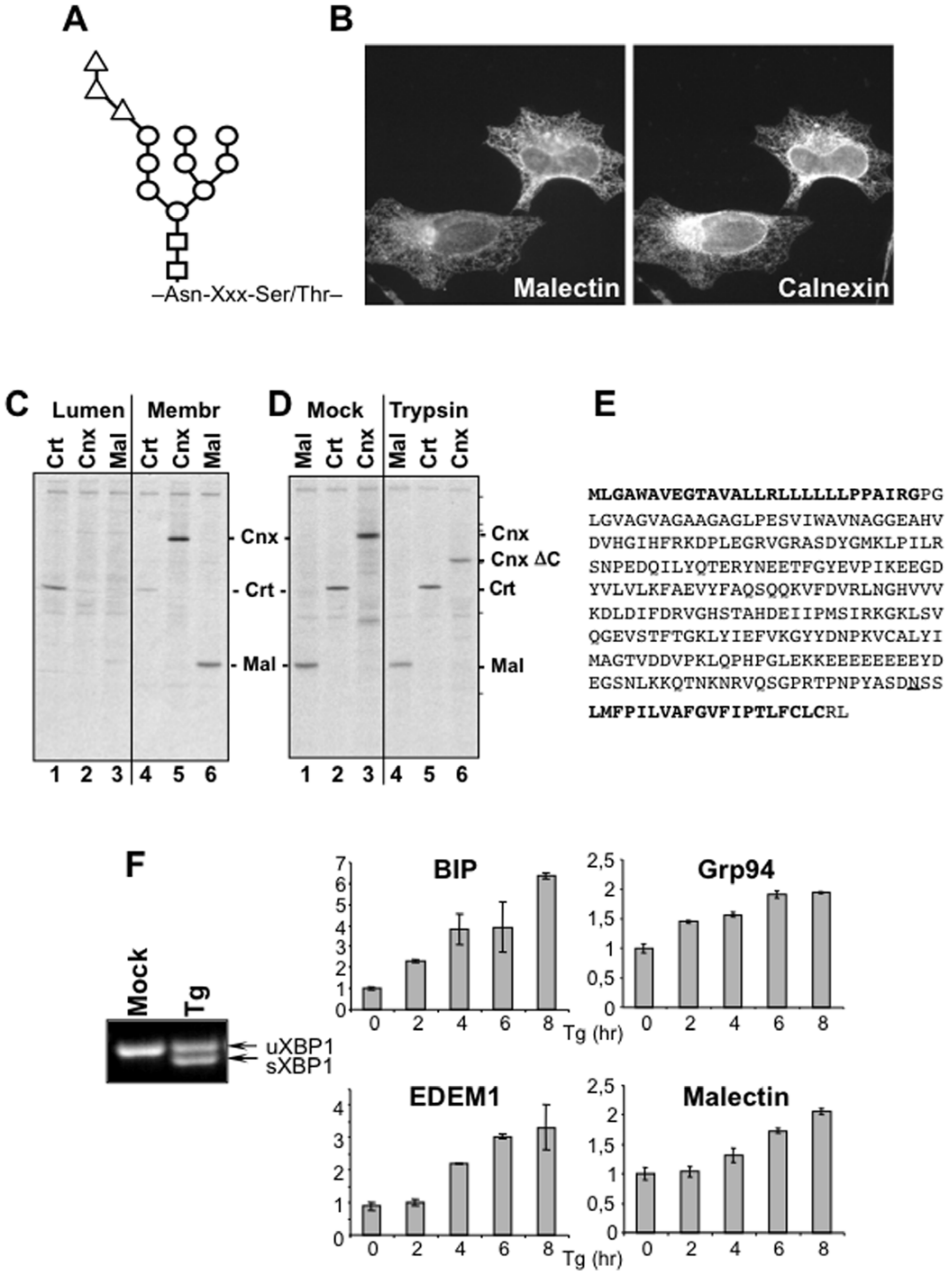
Subcellular localization, topology and ER stress induction of Malectin. **A** The asparagine-linked core oligosaccharide is composed of two N-acetylglucosamine (squares), nine mannose (circles) and three glucose residues (triangles). **B** Ectopically expressed Malectin-HA was visualized with an anti-HA antibody (left panel), Calnexin with a polyclonal antibody recognizing the endogenous protein (right panel). **C** Luminal *vs* membrane localization of ER chaperones was assessed after carbonate extraction of microsomes. **D** Intact microsomes were incubated with or without (Mock) trypsin to remove cytosolic portions of the labeled polypeptide chains. Calnexin ΔC lacks the 90 cytosolic residues. Molecular weight markers (200, 116, 97, 66, 45, 30 kDa) are shown on the right. **E** Human Malectin comprises 292 residues. The signal sequence and the transmembrane anchor are in bold, the putative N-glycosylation site is underlined. **F** Thapsigargin (Tg) triggers ER stress (uXBP1 and sXBP1 are unspliced and spliced XBP1 transcripts, respectively). Fold-induction of BiP, Grp94, EDEM1 and Malectin transcripts as determined by real time PCR (in triplicate). Actin served as negative control.

In Eukarya, pre-assembled glucose_3_-mannose_9_-N-acetylglucosamine_2_- oligosaccharides (**Fig. 1A**) are transferred from a lipid donor in the ER membrane onto Asn-X-Ser/Thr sequons of nascent polypeptide chains emerging in the ER lumen. The processing of protein-bound oligosaccharides determines the fate of the associated polypeptide chains. The intervention of several ER-resident sugar processing and sugar binding proteins in protein folding and quality control has been reported [1], [2]. Briefly, the translocon-associated α-glucosidase I removes the outermost glucose residue as soon as the oligosaccharide is covalently attached to the polypeptide chain emerging in the ER lumen. Immediately after, the soluble α-glucosidase II removes the second glucose. This generates a mono-glucosylated oligosaccharide that recruits the lectin chaperones Calnexin and Calreticulin as well as the oxidoreductase ERp57 [3], [4]. ERp57 catalyses formation of inter- and intramolecular disulfide bonds, a rate-determining step in polypeptide folding. The oxidative polypeptide folding in the Calnexin chaperone system starts co-translationally, as soon as the first native disulfide pair emerges into the ER lumen, and is concluded post-translationally [5]. The co-translational association of nascent glycopolypeptides with Calnexin and Calreticulin [6], [7], [8] shows that generation of the mono-glucosylated intermediate of the oligosaccharide processing might occur in a matter of few seconds. Native polypeptides released from the Calnexin chaperone system are de-glucosylated by the α-glucosidase II, exit the ER and are transported at their intra- or extracellular site of activity. However, if the folding program has not been completed, one terminal glucose residue is added-back by the folding sensor UDP-glucose:glycoprotein glucosyltransferase (UGT1) to prolong folding attempts in the Calnexin folding environment [9]. The removal of up to 4 terminal mannose residues by the ER mannosidase I and by EDEM proteins eventually interrupts unproductive retention in the folding environment and directs terminally misfolded polypeptides to dislocons at the ER membrane. Dislocons contain adaptor proteins and membrane-embedded E3 ubiquitin ligases that regulate substrate retro-translocation across the ER membrane for proteasomal degradation [10], [11], [12].

The recent discovery of Malectin, an ER-resident protein that binds oligosaccharides displaying terminal glucose residues with a strong preference for di-glucosylated ones *in vitro* [13], [14], [15], led to many speculations on a possible involvement of this lectin in the Calnexin chaperone system and in glycoprotein quality control in the mammalian ER [1], [2].

Here we show that Malectin is an ER stress-induced type I membrane protein that associates with newly synthesized glycoproteins in living cells. Analysis of the influenza virus HA revealed that Calnexin and Malectin had distinct kinetics of association with newly synthesized polypeptides and that Malectin preferentially associated with misfolded HA conformers. Changes in the intralumenal levels of Malectin did not affect the function of the Calnexin chaperone system or the maturation of HA, an *obligate* Calnexin substrate. It is therefore unlikely that Malectin participates in the Calnexin chaperone system. On the other hand, enhancement of Malectin expression to simulate ER-stress conditions resulted in prolonged Malectin association with two α1AT variants used here as model cargo glycoproteins. Even though we did not assess the oligosaccharide specificity of Malectin in the living cell, our data showed that Malectin association with glycoproteins might affect processing of N-linked oligosaccharides and might substantially reduce secretion of select cargo proteins.

## Results

### Malectin topology and properties

Malectin is an ER-resident protein as shown by its co-localization with the conventional ER marker Calnexin in indirect immunofluorescence (**Fig. 1B** and [14]). We first determined the membrane topology of Malectin because only the luminal orientation of the protein's carbohydrate binding-site would be consistent with a role of this protein in ER quality control. To this end, mammalian cultured cells were transfected with a plasmid for expression of HA-tagged human Malectin. Seventeen hours after transfection, cells were pulsed for 15 minutes with ^35^S-methionine and -cysteine to be then broken with 10–13 passages through a needle. After removal of nuclei and cytosol, the luminal content of endomembranes was separated from the membrane fraction upon carbonate extraction as described in [16]. Calreticulin, a marker of the ER lumen, was mainly immunoisolated from the luminal fraction (**Fig. 1C**, lane 1 *vs* 4). Calnexin, a marker of the ER membrane (lane 5 *vs* 2), and Malectin (lane 6 *vs* 3) were mainly immunoisolated from the membrane fraction.

To assess the orientation of Malectin in the ER, microsomes were mock-treated (**Fig. 1D**, lanes 1–3) or incubated for 1 hour at 37°C with trypsin to remove the cytosolic portion of microsomal proteins. Trypsin treatment did not modify the electrophoretic mobility of Malectin (**Fig. 1D**, lane 1 *vs* 4) and of Calreticulin (lane 2 *vs* 5). For Calreticulin, this was expected because Calreticulin is a luminal protein and remains therefore inaccessible to trypsin. For Malectin, this showed that the bulk of the polypeptide chain is located in the ER lumen. As a positive control, trypsin-treatment changed the electrophoretic mobility of Calnexin by removing the 90 cytosolic C-terminal residues of this type I protein (**Fig. 1D**, lane 6, Calnexin ΔC).

Consistent with these experimental data, the use of SignalP 3.0 [17] and PSORT II [18] algorithms to predict signal sequence *versus* transmembrane sequences in Malectin revealed a signal peptide from residues 1 to 27, a transmembrane region between residues 271–290 and a cytosolic tail comprising only two residues (**Fig. 1E** and [14]). Malectin contains a consensus sequence for N-glycosylation, which is not used, most likely because the acceptor asparagine residue (underlined in **Fig. 1E**) is adjacent to the transmembrane anchor.

### Malectin expression is induced upon ER stress

To determine whether the expression level of Malectin was enhanced under ER stress conditions, cultured cells were mock-treated or exposed to 300 ng/ml thapsigargin (Tg) for up to 8 hr before determining the level of BiP, EDEM1, Grp94 and Malectin transcripts by Real Time PCR. The splicing of Xbp1 transcripts confirmed activation of the unfolded protein response (**Fig. 1F**). The Real Time analysis revealed a 7-fold induction of BiP, a 3.5-fold induction of EDEM1 and a 2-fold induction of Grp94 and Malectin transcripts (**Fig. 1F**). Thus, Malectin expression is enhanced in response to accumulation of misfolded polypeptides in the ER lumen.

### Changes in Malectin levels do not affect biogenesis of influenza virus HA

**Figure 2:**
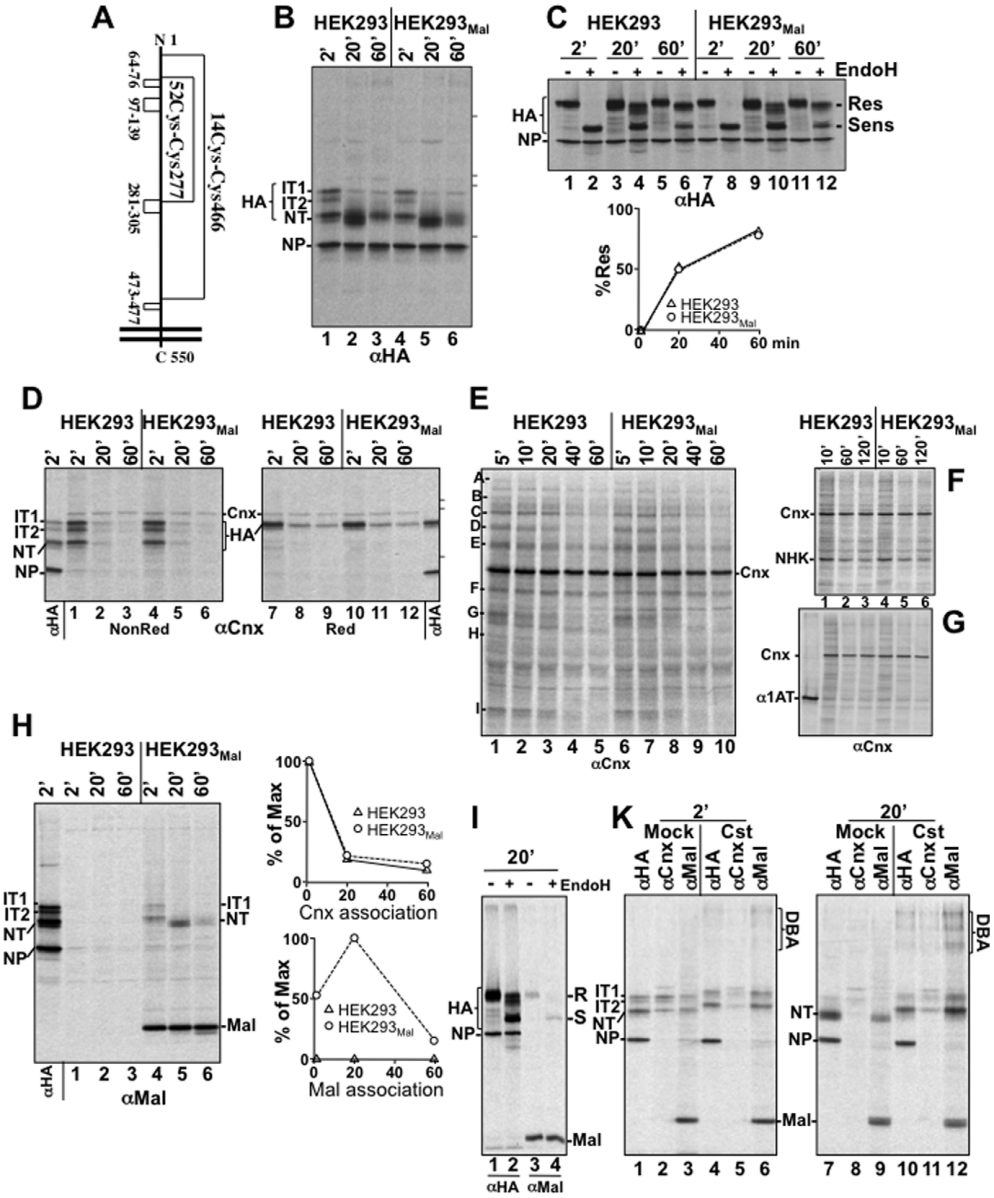
Consequences of Malectin overexpression on HA maturation. **A** Schematics showing the cysteine residues covalently linked in the native HA. **B** Influenza virus HA was immunoisolated from detergent lysates of influenza virus-infected cells with normal (lanes 1–3) or elevated levels of Malectin (stably transfected HEK293_Mal_ cells, lanes 4–6). The folding intermediates IT1, IT2 and NT are shown. The viral, non-glycosylated nuclear protein (NP) is also recognized by the antibody used. Molecular weight markers (200, 116, 97, 66 kDa) are shown on the right. **C** Assessment of EndoH-sensitivity of oligosaccharides displayed on HA in HEK293 (lanes 1–6) and in HEK293_Mal_ cells (lanes 7–12). Res, EndoH-resistant HA; Sens, EndoH-sensitive HA. The % of EndoH-resistant HA (Res in the upper panel) has been quantified. This gel is representative of a series of experiments in which HA maturation was monitored at different chase times. **D** Release of HA from Calnexin in HEK293 (lanes 1–3 (non reducing gel); lanes 7–9 (reducing gel)) and in HEK293_Mal_ cells (lanes 4–6 (non reducing); lanes 10–12 (reducing)). Calnexin and associated substrates have been immunoisolated from the lysate of 200'000 cells. Lane αHA shows the ratio of IT1, IT2, NT in infected cells after 2 min chase and the mobility of NP, which does not co-immunoprecipitate with Cnx (specificity control). Viral proteins in the lane αHA have been immunoisolated from the lysate of 20'000 cells. Molecular weight markers (116, 97, 66 kDa) are shown on the right. **E** Same as **D** for endogenous Calnexin substrates. Select polypeptides are shown with A–I. **F** Same as **D** for ectopically expressed NHK. **G** Same as **D** for ectopically expressed α1AT. **H** Same as **D** for Malectin-associated HA. The specificity of the anti-Malectin immunoprecipitation is confirmed by the low cross reactivity in infected cells that did not contain ectopically expressed Malectin (lanes 1–3) and by the lack of co-precipitation of the non-glycosylated NP (lane 1) with Malectin (lanes 4–6). Quantitations of HA release from Calnexin (upper panel, reducing gel, **Fig. 2D**)) and from Malectin (lower panel, gel in Fig. 2H) are shown. Ectopically expressed Malectin-HA and the associated influenza virus HA have been immunoisolated from detergent extracts with an antibody to the HA-tag sequence (YPYDVPDYA). This sequence is not present in the X-31 influenza virus HA (as confirmed by the lack of influenza virus protein in the immunoisolates of cells not expressing Malectin-HA, lanes 1–3). **I** Same as **C** for the labelled HA immunoisolated from cells after 20 min chase (lanes 1–2) and for Malectin-associated HA (lanes 3–4) after 20 min chase. **K** HA immunoisolated after 2 min chase from cells in the absence (Mock, lane 1) or in the presence of 1 mM Castanospermine (Cst, lane 4). The same for Calnexin-associated HA (Mock, lane 2; Cst, lane 5). The same for Malectin-associated HA (Mock, lane 3; Cst, lane 6). The analysis was also performed for cells solubilised after 20 min chase (lanes 7–12). Since Cst inhibits removal of glucose residues from HA-bound oligosaccharides, HA has slower electrophoretic mobility in lanes 4–6 and 10–12. The experiments shown in panels I–K have been performed in HEK293_Mal_ cells.

Influenza virus HA is a canonical model substrate used in the past 20 years to characterize the mechanisms regulating chaperone-assisted glycoprotein maturation in the ER of mammalian cells [4], [8], [19], [20], [21], [22]. HA acquires 6 native intramolecular disulfide bonds (**Fig. 2A**) in association with Calnexin, Calreticulin and ERp57. It is defined as an *obligate* client of the Calnexin chaperone system because any perturbation of the activity of α-glucosidases, Calnexin, Calreticulin, ERp57 or UGT1 results in substantial folding defects [19], [23], [24], [25]. HA is therefore an appropriate substrate to determine whether the glucose-binding lectin Malectin interferes with the function of Calnexin, which also associates with newly synthesized polypeptides displaying glucosylated oligosaccharides [4].

Four hours after infection with influenza virus, HEK293 cells were metabolically labeled and the viral polypeptides (the glycoprotein HA and the non-glycoprotein NP in **Fig. 2**) were immunoisolated from cell lysates after 2, 20 and 60 min chase in the absence of radioactivity.

Separation of the proteins in nonreducing gels, revealed three HA folding intermediates with different oxidation states after 2 min chase (**Fig. 2B**, lane 1; IT1 contains only small loop disulfides, IT2 also contains the Cys52-Cys277 disulfide, NT is fully oxidized [21]). The ratio between incompletely (IT1+IT2) and fully oxidized HA (NT) was 3∶5 indicating that oxidative HA folding was still in progress. After 20 min chase, the oxidative phase of HA folding had virtually been completed and most of the labeled HA migrated as a single polypeptide band in nonreducing gels (NT, **Fig. 2B**, lane 2). After 60 min chase (lane 3), the amount of radio-labeled HA immunoisolated from HEK293 cells decreased because the protein enters in membranes poorly soluble in CHAPS, the zwitterionic detergent used in these experiments to preserve chaperones:HA complexes (see below), and because viral particles containing labeled HA are released from infected cells. Efficient maturation of HA was confirmed by the progressive attainment of EndoH-resistant oligosaccharides that occurs when native HA traverses the Golgi compartment (**Fig. 2C**, lanes 1–6).

Repetition of the experiments in HEK293 cells stably expressing elevated levels of Malectin (HEK293_Mal_) failed to reveal significant differences. After 2 min chase, infected HEK293_Mal_ cells contained the same ratio of incompletely (IT1+IT2) *vs* fully oxidized HA (NT, **Fig. 2B**, lane 4) as HEK293 cells. After 20 min chase, HA oxidation was virtually complete (lane 5) and the attainment of EndoH-resistant oligosaccharides also progressed as in cells expressing normal levels of Malectin (**Fig. 2C**, lanes 7–12). Likewise, the reduction of the intralumenal level of Malectin upon specific RNA interference did not affect oxidative HA maturation (**Fig. S1**). The finding that variations in the intralumenal level of Malectin do not affect HA folding implies that Malectin is not an essential component of the Calnexin chaperone system whose unperturbed function is required for productive HA maturation [19], [23], [24], [25].

### Malectin does not affect oxidative folding of Calnexin-associated HA

To confirm that Malectin overexpression does not affect the function of the Calnexin chaperone system, Calnexin and the associated HA conformers were immunoisolated from HEK293 and HEK293_Mal_ cell lysates. Analysis in nonreducing gels showed that after 2 min chase, all three HA folding intermediates were associated with Calnexin in HEK293 cells (**Fig. 2D**, lane 1). The ratio (IT1+IT2):NT was 5∶3, thus confirming the preferential association of incompletely oxidized HA folding intermediates with the lectin chaperone [4], [5], [19], [25]. After 20 min chase, oxidative HA folding had virtually been completed (**Fig. 2B**, lane 2). Consistently, Calnexin had released most of the labeled HA (**Fig. 2D**, lane 2). HA release from Calnexin is better appreciated in reducing gels, in which IT1, IT2 and NT migrate as a single polypeptide band (HA in lanes 7–9). In HEK293_Mal_ cells, the same amount of radio-labeled, newly synthesized HA did associate with Calnexin (**Fig. 2D**, compare lane 4 with 1 in the nonreducing gel and lane 10 with 7 in the reducing gel). This showed that high levels of luminal Malectin do not interfere with HA association with Calnexin. Malectin overexpression did also not affect the ratio of (IT1+IT2):NT transiently associated with Calnexin (compare lane 4 with 1), or the kinetics of HA release from Calnexin during the chase (compare lanes 10–12 with 7–9, and quantitation in **Fig. 2H**, upper panel).

### Variations in the intralumenal level of Malectin do not affect substrate association with Calnexin

Unperturbed Calnexin function upon Malectin overexpression was not a peculiarity of cells infected with influenza virus. In fact, the amount of labeled endogenous polypeptides associating with Calnexin was essentially the same in HEK and in HEK293_Mal_ cells (compare lane 1 with 6 in **Fig. 2E**). The kinetic of release from Calnexin of endogenous (**Fig. 2E**, lanes 1–5 *vs* 6–10) and of ectopically expressed substrates (NHK (**Fig. 2F**) and α1AT (**Fig. 2G**)) did also not vary upon elevation of the intralumenal Malectin levels. Likewise, the reduction of the intralumenal level of Malectin obtained upon RNA interference did not affect association and kinetic of release from Calnexin of model glycoproteins (**Fig. S1**). Thus, even though it binds glucosylated oligosaccharides [13], [14], [15], Malectin does not compete with Calnexin for association with newly synthesized polypeptides.

### Malectin and Calnexin association with HA conformers

Next, we determined whether Malectin associates with influenza virus HA. Analysis of the immunoprecipitates revealed a transient association of Malectin with HA with a peak of binding after 20 min chase (**Fig. 2H**, lanes 4–6 and quantitation, lower panel), when Calnexin had already released most of the newly synthesized glycoprotein (**Figs. 2D and 2H**, quantitation, upper panel). Analysis of the nonreducing gels showed that, unlike Calnexin (**Fig. 2D**, lanes 1 and 4), Malectin preferentially associated with the fully oxidized NT and less abundantly with the partially oxidized HA conformers after 2 min chase (**Fig. 2H**, lane 4). Association of extensively oxidized HA conformers persisted during the chase and occurred in the ER because Malectin is localized in the ER (**Fig. 1**) and because it associated with the fraction of cellular HA (**Fig. 2I**, lanes 1–2) displaying EndoH-sensitive oligosaccharides (lanes 3–4). The delayed kinetics of binding (peak at 2 min for Calnexin and at 20 min for Malectin, **Fig. 2H**) and the association of Malectin with more extensively oxidized HA conformers revealed that Malectin and Calnexin do associate with distinct HA populations.

### HA misfolding enhances Malectin binding

The delayed association of Malectin with extensively oxidized HA conformers retained in the ER (**Figs. 2H-2I**) led us to hypothesize that Malectin might associate with a fraction of HA characterized by slow attainment of the native structure or with non-native HA. To check whether HA association with Malectin is enhanced under conditions that inhibit HA maturation, influenza virus infected cells were exposed to castanospermine (Cst). Cst inhibits generation of mono-glucosylated oligosaccharides and drastically reduces substrate association with Calnexin (**Fig. 2K**, lane 2 *vs* 5 and [4], [20]). Under these conditions, HA maturation is substantially impaired as shown by the aberrant formation of HA-containing disulfide-bonded complexes, which are retained in the ER lumen upon association with the luminal chaperone BiP [4], [19], [20], [25], [26]. Our data confirmed that cell exposure to Cst results in progressive accumulation of HA-containing disulfide-bonded aggregates (DBA), which are already visible after 2 min chase in the presence of Cst (DBA in **Fig. 2K**, lane 4 *vs* 1) and become more abundant after 20 min chase (lane 10 *vs* 7). Analysis of the immunocomplexes containing Malectin and HA revealed stronger association in cells exposed to Cst (lane 6 *vs* 3 after 2 min chase and lane 12 *vs* 9 after 20 min chase). More importantly, the co-precipitation of the high molecular weight HA-containing DBA (lanes 6 and 12 in **Fig. 2K**) revealed that Malectin contributes with BiP [25], [26] as a crucial ER retention factor for misfolded HA.

### Malectin overexpression and cargo retention in the ER lumen

HA is highly efficient folder and Malectin overexpression has no substantial consequence on its maturation. However, the finding that Malectin could be involved in ER-retention of folding-defective/misfolded HA chains led us to assess whether Malectin overexpression affects secretion of glyco-polypeptides characterized by slower, less efficient and less Calnexin-dependent maturation. We therefore selected two α1AT variants as model cargo proteins to be expressed in cells with variable Malectin content. The Null Hong Kong variant of α1AT (NHK [27]) is mostly degraded from the ER when ectopically expressed in sub-confluent mammalian cultured cells [28] but, independent of its expression level, about 20% of the synthesized protein is secreted in the extracellular media [29]. Secretion of α1AT is more efficient and reaches about 60% of the polypeptide chains entering the ER.

**Figure 3:**
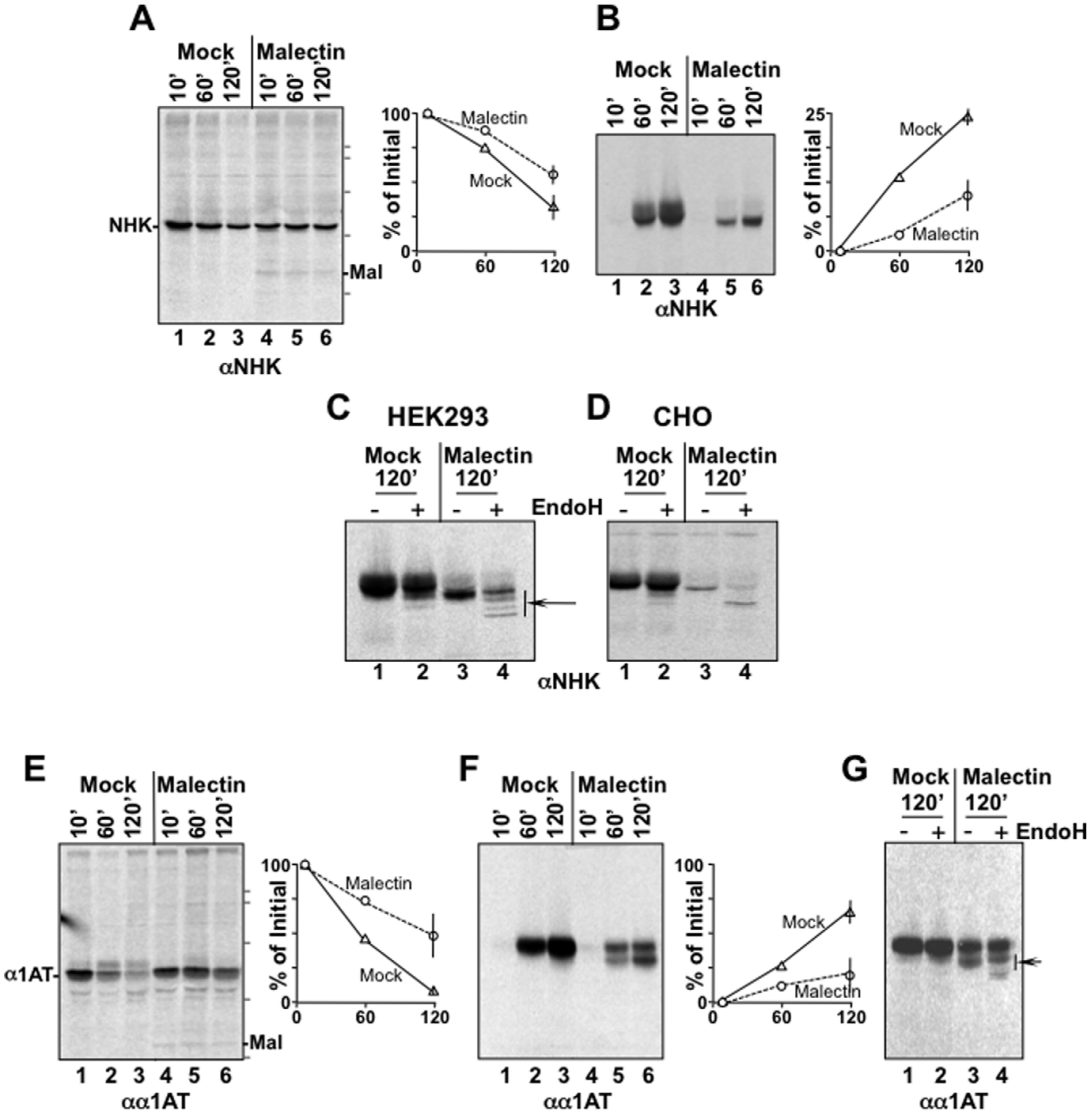
Malectin overexpression interferes with oligosaccharide processing and cargo protein secretion. **A** Intracellular, labelled NHK in HEK293 cells (lanes 1–3) and in HEK293 cells co-transfected with a plasmid for expression of Malectin-HA (lanes 4–6). Malectin co-precipitates with NHK throughout the chase (lanes 4–6). Molecular weight markers (116, 97, 66, 45, 30 kDa) are shown on the right. Quantitations in panels A, B, E, G are for at least two independent experiments (error bars represent SD). Data have also been reproduced in other cell lines (CHO cells in panel **D**, HeLa cells transiently transfected for Malectin overexpression and HEK293_Mal_, not shown). **B** Same as **A** for secreted NHK. **C** Assessment of EndoH-sensitivity of oligosaccharides displayed on NHK secreted from HEK293 cells (lanes 1–2) or from HEK293 cells co-transfected for expression of Malectin-HA (lanes 3–4). The arrow shows the fraction of labelled NHK displaying EndoH-sensitive oligosaccharides in lane 4. **D** Same as **C** for NHK secreted from CHO cells. **E**–**G** Same as **A**–**C** for α1AT. Molecular weight markers (116, 97, 66, 45 kDa) are shown on the right.

HEK293 cells were transiently transfected with a plasmid for expression of NHK (**Fig. 3A**, lanes 1–3) or co-transfected for expression of NHK and human Malectin (lanes 4–6). Seventeen hours later, cells were metabolically labeled, chased for 10, 60 or 120 min and ectopically expressed NHK was immunoisolated from cell lysates and separated in SDS-PAGE. Like HA and α1AT (**Figs. 2** and **Fig. 3E**, respectively), analysis of the labeled NHK after a 10 min chase revealed the same electrophoretic mobility in cells expressing normal (**Fig. 3A**, lane 1) and elevated levels of Malectin (lane 4), consistent with unperturbed N-glycosylation of nascent polypeptides. In the experiment shown in the figure, after 120 min chase, mock-transfected cells contained 36% of the initial amount of labeled NHK (**Fig. 3A**, lane 3 *vs* 1). The amount of residual intracellular NHK at the end of the chase was increased to 57% in cells characterized by elevated Malectin levels (**Fig. 3A**, lane 6 *vs* 4). In these cells, Malectin did co-precipitate, throughout the chase, with the labeled NHK retained intracellularly showing a long-lasting association between the two proteins (-Mal in **Fig. 3A**, lanes 4–6).

### Malectin overexpression interferes with NHK secretion

NHK secretion was monitored by immunoisolation of the labeled protein from the extracellular media harvested at the end of each chase time. After a 10 min chase, which is insufficient for a secretory protein to exit the cellular secretory line, the media of mock-transfected (**Fig. 3B**, lane 1) and of Malectin over-expressing cells (lane 4) did not contain labeled NHK. However, with progression of the chase, the amount of NHK secreted from wild type cells increased to reach the 24% of the synthesized protein (**Fig. 3B**, lane 3). Consistent with the delay in disappearance of radiolabeled NHK from the intracellular milieu (**Fig. 3A**, lanes 4–6), overexpression of Malectin resulted in a drastic decrease of NHK secretion to less than 10% of the synthesized protein (**Fig. 3B**, lane 6).

### Malectin overexpression results in secretion of NHK displaying partly EndoH-sensitive N-glycans

Comparison of the electrophoretic mobility of NHK secreted from wild type cells (**Fig. 3B**, lanes 2–3) or from cells expressing high levels of Malectin (lanes 5–6), revealed a slightly faster mobility of the latter polypeptide, a possible symptom of aberrant processing of the NHK-bound oligosaccharides. Normally, N-linked glycans displayed on secreted proteins are extensively modified in the Golgi compartment to become resistant to EndoH cleavage [30]. This was in fact observed for the NHK protein secreted from wild type cells that remained fully glycosylated and conserved the same gel mobility upon mock- and upon EndoH-treatment (**Fig. 3C**, lanes 1 and 2, respectively). Instead, the NHK secreted from cells expressing high levels of Malectin was partially de-glycosylated upon EndoH-treatment, as shown by the generation of labeled polypeptide bands with faster electrophoretic mobility upon exposure of the polypeptide to the endoglycanase (**Fig. 3C**, arrow in lane 4). Thus, Malectin overexpression reduced NHK secretion and resulted in secretion of NHK displaying aberrant, partly EndoH-sensitive N-glycans.

### Malectin overexpression interferes with efficient protein de-glucosylation in the ER

Interestingly, inefficient attainment of EndoH-resistant oligosaccharides occurs when glycoproteins are released from the ER before complete oligosaccharide de-glucosylation. Under these circumstances, a salvage pathway must be activated in which terminal glucoses must be removed by a Golgi-resident endo-α-D-mannosidase before complex glycosylation can proceed [31], [32]. We reasoned that Malectin binding to glucosylated oligosaccharides [13], [14], [15] could interfere with the activity of ER α-glucosidases thus resulting in unconventional release from the ER of glucosylated polypeptides. To assess this, we repeated the same experiments described above in Chinese hamster ovary (CHO). CHO cells lack the Golgi-resident endo-α-D-mannosidase. In these cells, release of glucosylated oligosaccharides from the ER would be revealed by secretion of proteins displaying EndoH sensitive glycans [33]. Like in HEK293 cells (**Fig. 3C**), the NHK normally secreted from CHO cells displayed EndoH-resistant oligosaccharides (**Fig. 3D**, lanes 1–2). However, when Malectin was overexpressed, the vast majority of secreted NHK displayed EndoH-sensitive oligosaccharides (compare lane 4 in **Fig. 3D** with lane 4 in **Fig. 3C**). We therefore concluded that Malectin associates with glucosylated oligosaccharides in the ER lumen thereby interfering with their efficient de-glucosylation.

### Malectin inhibits secretion of α1AT

The experiments with α1AT essentially confirmed the data shown above. Malectin did co-precipitate with α1AT throughout the chase thereby increasing the fraction of newly synthesized α1AT retained intracellularly (from 14 to 47% in the experiment shown in **Fig. 3E**, lanes 1–3 *vs* 4–6). Enhanced intracellular retention corresponded to a substantial reduction of the secretion of the labeled protein (from 65 to 18% in **Fig. 3F**, lanes 1–3 *vs* 4–6). Moreover, the α1AT secreted from cells containing high levels of Malectin was separated in two distinct polypeptide bands (**Fig. 3F**–**3G**) displaying aberrant, partially EndoH-sensitive oligosaccharides (arrow in **Fig. 3G**, lane 4). This confirmed that Malectin overexpression interferes with normal processing of oligosaccharides displayed on secretory cargo proteins and reduces cargo protein secretion.

## Discussion

### Malectin, a novel ER lectin

Malectin is a novel sugar binding, ER-resident protein highly conserved in Metazoa. It shares structural similarity with carbohydrate binding modules (CBM) of prokaryotic glycosyl hydrolases. In carbohydrate microarray analyses, Malectin binds maltose, nigerose and related di-glucosides [13], [14], [15]. Amongst lipid-linked high mannose oligosaccharides, Malectin shows a strong preference for glucose**_2_**-mannose**_7_**-N-acetylglucosamine**_1_**-. The much lower association of Malectin with glucose**_3_**-mannose**_7_**-N-acetylglucosamine**_1_**- and glucose**_1_**-mannose**_9_**-N-acetyl glucosamine**_2_**- species might be ascribed to the presence of trace contaminants in the glycans isolated from drug-treated cells [14]. In any case, glucosylated glycan structures are present in the ER lumen of living cells as lipid-linked precursors in the ER membrane and, after the intervention of the oligosaccharyl transferase, as protein-bound tags that determine the fate of the associated polypeptide [34], [35]. For these reasons, the discovery of Malectin led to many speculations on a possible involvement of this lectin in the Calnexin chaperone system and in glycoprotein quality control in the mammalian ER [1], [2].

### Malectin associates with newly synthesized glycoproteins in the ER

The topology of Malectin reported in this study, with the ectodomain containing the putative lectin site in the ER lumen, is consistent with a role of Malectin in glycoprotein quality control. Our data show that N-glycosylation of newly synthesized polypeptides progresses normally upon Malectin overexpression. We therefore consider unlikely that Malectin binds partially or fully glucosylated lipid-linked oligosaccharides at the ER membrane, as this would interfere with oligosaccharide synthesis and/or with oligosaccharide transfer onto nascent polypeptide chains. Rather, our data indicate that Malectin associates with glycoproteins in the ER lumen. In fact, we report the immunoisolation from cell lysates and the characterization of complexes containing Malectin and newly synthesized HA retained in the ER as well as the detection of complexes containing Malectin and two soluble cargo glycoproteins, NHK and α1AT.

### Malectin does not compete with Calnexin for substrate binding

Malectin does associate with glucosylated oligosaccharides *in vitro* [13], [14], [15] and binds newly synthesized glycoproteins in living cells possibly affecting the efficiency of oligosaccharides de-glucosylation. However, our initial hypothesis that Malectin participates in the Calnexin chaperone system was not confirmed by experimental data. In fact, changes in the Malectin's intralumenal level did not affect the function of the “glucose-based” Calnexin chaperone system (**Figs. 2D, 2E–2G**, **Fig. S1**). This is testified by i) the equal amount of newly synthesized cellular and viral glycoproteins associating with Calnexin, ii) the unchanged kinetics of substrate release from Calnexin and iii) the unperturbed maturation of HA (an *obligate* client of the Calnexin chaperone system) in cells with low, normal or elevated levels of Malectin. In the case of HA, Calnexin and Malectin showed clearly different kinetics of binding with a sharp peak in the early phases after HA synthesis for Calnexin and a broader peak with a maximal association about 20 min after synthesis for Malectin. Calnexin and Malectin associated with distinct HA conformers. In particular, Malectin showed persistent binding to extensively oxidized ER-retained HA, which poorly associates with Calnexin. It is possible that Malectin traps the minor fraction of HA that fails to rapidly attain the native structure in the Calnexin chaperone system. Certainly, it retains disulfide-bonded conformers expressed under conditions that inhibit HA maturation.

### Malectin interferes with oligosaccharide processing and protein secretion

Analysis of the model cargo proteins α1AT and NHK, which are characterized by slower, less efficient and less Calnexin-dependent maturation compared to HA revealed that Malectin overexpression may interfere with oligosaccharide processing and secretion of select glycoproteins. The finding that the intervention of the Golgi-resident endo-α-D-mannosidase was required for conversion of NHK oligosaccharides to EndoH-resistant structures is consistent with a model claiming that by binding glucosylated structures as established *in vitro* [13], [14], [15], Malectin may interfere with the activity of the ER-resident α-glucosidases thereby reducing the efficiency of protein de-glucosylation. The Golgi endo-mannosidase intervenes in fact under these circumstances to remove terminal glucose residues and to allow complex glycosylation by Golgi-resident glycosyl transferases [31], [32], [33], [36]. Appropriate sugar processing in the ER is a pre-requisite for efficient cargo binding to cargo lectins that regulate ER export of a subset of secretory proteins. It has been reported that the presence of terminal glucose residues substantially inhibits cargo protein association with cargo lectins [37]. It is therefore possible that defective de-glucosylation and persistent Malectin association contribute to a selective inhibition of the export of those glycoproteins that rely on cargo lectins for secretion [38]. The proposed interference of Malectin with the activity of ER-resident α-glucosidases does not affect, as far as we can tell, the association and the release of glycoproteins (of viral, ectopic or endogenous origin (**Figs. 2D**–**2G**)) with Calnexin. One possibility is that Calnexin and Malectin participate in two distinct chaperone systems operating in parallel in the ER lumen. One could speculate that upon overexpression, Malectin only determines the fate of the fraction of a given polypeptide that enters the *alternative* chaperone pathway. For HA, this fraction is very low because 3 oligosaccharides positioned at the polypeptide N-terminus elicit co-translational entrance of nascent chains in the Calnexin chaperone system [5], [6], [8] and exclude intervention of alternative pathways [8]. For nascent glycoproteins with N-glycans late in the sequence (e.g. the α1-antitrypsin variants analyzed here), partitioning in alternative chaperone pathways is more likely to occur [8]. For several ER-resident lectins such as Calnexin/Calreticulin [39] and EDEM1 [40], the lectin function is dispensable for association with misfolded conformers. It seems therefore relevant to establish the role of the lectin site of Malectin for the *in vivo* function of this novel ER-resident quality control factor. Since Malectin is a stress-responsive gene, it is conceivable that Malectin-regulated inhibition of cargo export is activated under conditions of ER stress, possibly to avoid that defective gene products are secreted under condition of aberrant functioning of the ER quality control machineries.

## Materials and Methods

### Real Time PCR

For analysis of ER-stress induced gene transcription, cells were plated in 6-cm dishes without (Mock) or with thapsigargin 300 nM. After 0-2-4-6-8 h cells were lysed in TRIzol reagent (Invitrogen) and RNA isolated according to the instructions of the manufacturer. Quantitative PCR was performed using 7900HT Fast Real-Time PCR System. The PCR reaction mixtures were performed in a 20 µl final volume, containing 10 µl Power SYBR Green PCR master mix (Applied Biosystem), 5 µl cDNA, 4 µl dH_2_O and 1 µl primer mix (0.5 µM final). The housekeeping gene β-actin was used as reference. Data were analyzed using the SDS 2.2.2 software. The primers were as follows: β-actin, CTTCCTGGGCATGGAGTCCT (for) and GGAGCAATGATCTTGATCTT (rev); BiP, GAGTTCTTCAATGGCAAGGA (for) and CCAGTCAGATCAAATGTACCC (rev); EDEM1, AAGATTCCACCGTCCAAGTC (for) and GTATCATTGC TCCGGAGGTT (rev); Malectin, GACTATGGCATGAAACTGCC (for) and GACTGTGCAAAGTAGACCTC (rev); Grp94, CTGGGACTGGGAACTTATGAATG (for) and TCCATATTCGTCAAACAGACCAG (rev); spliced and unspliced XBP1, AAACAGAGTAGCAGCTCAGACTGC (for) and TCCTTCTGGGTAGACCTCTGGGA (rev).

### Expression plasmids, antibodies and inhibitors

Plasmids and antibodies for the α1-antitrypsin variants are described in [41], [42]. A plasmid for expression of HA-tagged Malectin was prepared by amplifying the protein coding sequence from the full-length cDNA clone, product IRAUp969B111D (Imagenes) with the primers CACCGAATTCCCACCATGCTGGGAGCCTGGGC and CCCACCCTCGAGTCA CAACCGGCAGAGGCAGAA. The construct was cloned upstream to a sequence coding for a HA-tag in a pcDNA3 vector for expression in mammalian cells. The monoclonal anti-HA and the polyclonal anti-α1 antitrypsin were from Sigma, the polyclonals anti-Calreticulin and anti-Calnexin (N-terminus) were from Stressgen. The sequence of the HA-tag at the Malectin C-terminus (YPYDVPDYA) is not present in the X-31 influenza virus HA. Ectopically expressed Malectin was also immunoprecipitated with a specific polyclonal antibody from Sigma.

### Cell Lines, transient transfections

HEK293 and CHO cells (from ATTC) were grown in appropriate culture media supplemented with 10% FBS. Cells at 80–90% confluence in a 6 cm tissue culture plate were transfected with the expression plasmid of interest (4 µg for single transfections, 6 µg total DNA for double transfections) using Lipofectamine 2000 (Invitrogen) according to the manufacturer instructions. Experiments were normally performed 17 hours after transfection.

### RNA interferences

For siRNA-based interference, HeLa cells (from ATTC) at 50% confluence in a 3.5 tissue culture plate were transfected with siRNA duplex (Ambion Inc, 50 pmol/dish) using Lipofectamine 2000 according to the manufacturer instructions. Four hours after transfection, the medium was replaced with MEM Alpha supplemented with 1% of non-essential amino acids (GIBCO). Thirty hours after siRNA transfection, cells were transfected with the expression plasmids of interest. Experiments were performed 48 hours post-siRNA transfection. siRNA targeting sequences for Malectin down-regulation: AGCUCACGAUGAAAUUAUA.

### Metabolic labelling, immunoprecipitations, immunoblots and analysis of data

Eighteen hours after transfection (or four hours after infection with the X-31 strain of influenza virus), cells were starved for 20 min in Met/Cys free medium, pulsed for 10 min with 50 µCi [^35^S]Met/Cys and chased for the indicated times with MEM Alpha supplemented with 5 mM cold Met/Cys. Postnuclear supernatant (PNS) was prepared by solubilization of cells in 400 µl/3,5 cm dish (or 800 µl/6 cm dish) ice-cold 2% CHAPS (Anatrace) in HEPES-buffered saline (HBS), pH 6.8, containing 20 mM N-ethylmaleimide and protease inhibitors. CHAPS-insoluble material was separated by centrifugation at 10'000 g for 10 min. Immunoprecipitations were performed by adding protein A beads (Sigma; 1∶10, w/v swollen in HBS) with the selected antibody and incubated for 2 h at 4°C. Immunoprecipitates were extensively washed (3×10 min) with 0.5% CHAPS in HBS, resuspended in sample buffer, boiled for 5 min and finally separated in SDS-PAGE. Gels were exposed to BioMax (Kodak) films and scanned with an Agfa scanner. Relevant bands were quantified by ImageQuant software (Molecular Dynamics). Immunoblots were performed using the SNAP i.d. protein detection system (Millipore). All primary antibodies were used at 1∶200–1∶333 dilutions. Secondary antibodies were HRP-conjugated and used at 1∶10'000 dilutions. The ECL-Plus detection system was from Amersham.

### Subcellular Fractionation and Preparation of Membranes Versus Luminal Content

Metabolically labeled cells were extensively washed in a 10-cm dish with isotonic buffer. They were gently detached with a rubber policeman and resuspended in 800 µl of homogenization buffer (10 mm triethanolamine, 10 mm acetic acid, 250 mm sucrose, 20 mm *N*-ethylmaleimide, 1 mm EDTA, and a mixture of protease inhibitors, pH 7.4). Cells were broken with 10–13 passages through a 22-gauge 1¼ needle. Postnuclear supernatants were subjected to a first ultracentrifugation (100,000 × *g* in TLA 120.2) to separate endomembranes from the cytosol. The endomembrane-containing pellet was washed twice and left for 25 min on ice in 500 µl of 100 mm Na_2_CO_3_ for carbonate extraction of the luminal content. After an additional ultracentrifugation step (100,000 × *g* in TLA 120.2), the luminal fraction was harvested and supplemented with antibodies to Cnx, Crt, or to HA-Malectin to determine their presence. The endomembrane fraction was subjected to an additional washing step with 100 mm Na_2_CO_3_ to minimize luminal contaminations and then resuspended in 800 µl of lysis buffer (2% CHAPS). The lysate was subjected to ultracentrifugation to eliminate insoluble material and the supernatant was analyzed by immunoprecipitation for chaperone content.

### Immunofluorescence Microscopy

2×10^5^ cells expressing Malectin-HA were seeded in a 6 cm Petri dishes containing 1% alcian blue treated glass cover slips. Cover slips were rinsed with PBS, and cells were fixed in 3,7% formaldehyde. After 2 short washings with 10 mM Hepes serum-free medium, and two additional washing with PBS, the antigen accessibility was improved by a 20-min incubation with 0,05% saponin, 10% Goat serum, 10 mM Hepes, 15 mM glycine. The monoclonal anti-HA (Sigma), the polyclonal anti-Calnexin), the Alexa 488-labeled goat anti-rabbit and the Alexa 594-labeled goat anti-mouse antibodies were diluted (1∶100) in the latter solution. Images were viewed on a Nikon eclipse E-800 fluorescent microscope, captured by a Hamamatsu EM-CCD Digital camera C9100 and analysed with the Open *lab* 3 software (Improvision, Inc., Lexington, MA).

## Supporting Information

Figure S1 **Malectin down-regulation does not affect HA maturation or NHK secretion.** **A** Malectin down-regulation by specific RNA interference. **B** Influenza virus HA was immunoisolated from detergent lysates of influenza virus-infected cells with normal (lanes 1–3) or reduced levels of Malectin (RNAi, lanes 4–6). **C** Release of Influenza virus HA from Calnexin in cells with normal (lanes 1–3) or reduced levels of Malectin (RNAi, lanes 4–6) analyzed in a reducing gel. **D** Assessment of EndoH-sensitivity of oligosaccharides displayed on HA expressed in cells with normal (lanes 1–6) or reduced (lanes 7–12) levels of Malectin. Res, EndoH-resistant HA; Sens, EndoH-sensitive HA. **E** Efficiency of Malectin down-regulation upon specific RNAi. **F** Disappearance of labeled NHK from control HEK293 cells (lanes 1–3) and from HEK293 cells expressing reduced levels of Malectin (lanes 4–6). **G** NHK secretion from control cells (lanes 1–3) and from cells expressing reduced levels of Malectin (lanes 4–6). **H** Release of ectopically expressed NHK and labeled endogenous proteins from Calnexin.
(TIF)
