# Supplementary figures and images for: Malectin Participates in a Backup Glycoprotein Quality Control Pathway in the Mammalian ER
Source: PLoS One. 2011 Jan 26;6(1):e16304. doi: 10.1371/journal.pone.0016304 (PMC3027649; doi:10.1371/journal.pone.0016304)

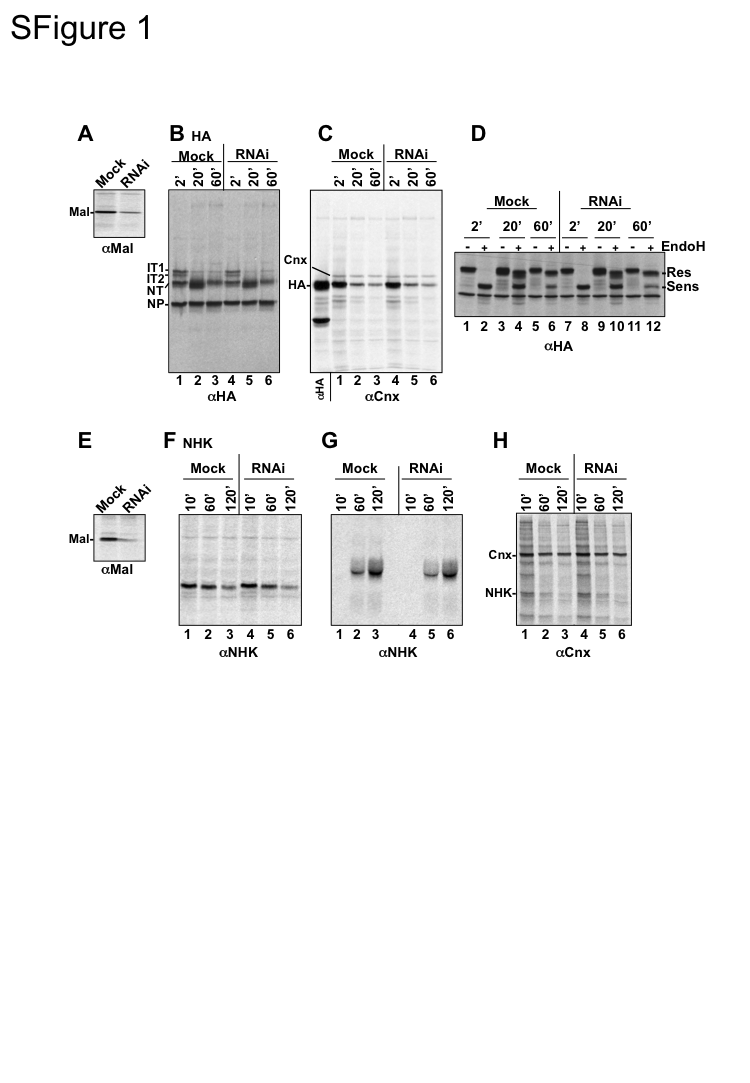

Supplement: Figure S1 — Malectin down-regulation does not affect HA maturation or NHK secretion. A Malectin down-regulation by specific RNA interference. B Influenza virus HA was immunoisolated from detergent lysates of influenza virus-infected cells with normal (lanes 1–3) or reduced levels of Malectin (RNAi, lanes 4–6). C Release of Influenza virus HA from Calnexin in cells with normal (lanes 1–3) or reduced levels of Malectin (RNAi, lanes 4–6) analyzed in a reducing gel. D Assessment of EndoH-sensitivity of oligosaccharides displayed on HA expressed in cells with normal (lanes 1–6) or reduced (lanes 7–12) levels of Malectin. Res, EndoH-resistant HA; Sens, EndoH-sensitive HA. E Efficiency of Malectin down-regulation upon specific RNAi. F Disappearance of labeled NHK from control HEK293 cells (lanes 1–3) and from HEK293 cells expressing reduced levels of Malectin (lanes 4–6). G NHK secretion from control cells (lanes 1–3) and from cells expressing reduced levels of Malectin (lanes 4–6). H Release of ectopically expressed NHK and labeled endogenous proteins from Calnexin. (TIF) [file pone.0016304.s001.tif]
